# Yes, You Can? A Speaker’s Potency to Act upon His Words Orchestrates Early Neural Responses to Message-Level Meaning

**DOI:** 10.1371/journal.pone.0069173

**Published:** 2013-07-24

**Authors:** Ina Bornkessel-Schlesewsky, Sylvia Krauspenhaar, Matthias Schlesewsky

**Affiliations:** 1 Department of Germanic Linguistics, University of Marburg, Marburg, Germany; 2 Research Group Neurotypology, Max Planck Institute for Human Cognitive and Brain Sciences, Leipzig, Germany; 3 Department of English and Linguistics, Johannes Gutenberg-University, Mainz, Germany; University of Goettingen, Germany

## Abstract

Evidence is accruing that, in comprehending language, the human brain rapidly integrates a wealth of information sources–including the reader or hearer’s knowledge about the world and even his/her current mood. However, little is known to date about how language processing in the brain is affected by the hearer’s knowledge about the speaker. Here, we investigated the impact of social attributions to the speaker by measuring event-related brain potentials while participants watched videos of three speakers uttering true or false statements pertaining to politics or general knowledge: a top political decision maker (the German Federal Minister of Finance at the time of the experiment), a well-known media personality and an unidentifiable control speaker. False versus true statements engendered an N400 - late positivity response, with the N400 (150–450 ms) constituting the earliest observable response to message-level meaning. Crucially, however, the N400 was modulated by the combination of speaker and message: for false versus true political statements, an N400 effect was only observable for the politician, but not for either of the other two speakers; for false versus true general knowledge statements, an N400 was engendered by all three speakers. We interpret this result as demonstrating that the neurophysiological response to message-level meaning is immediately influenced by the social status of the speaker and whether he/she has the power to bring about the state of affairs described.

## Introduction

“Mr. Gorbachev, tear down this wall!” This famous quote from former US-president Ronald Reagan’s speech in Berlin on June 12th 1987 has been hailed as “the most memorable delivered by any American president in the last quarter-century” [1]. Clearly, the impact of these words depends not only on the content of the message, but also on the speaker: had they been uttered by an ordinary citizen rather than by the president of the United States, their influence would have been lost. Reagan’s famous sentence thus provides a telling example of how the content of what is said (the message) is inextricably intertwined with who is saying it (the speaker). Here, we demonstrate using electrophysiological measures that this holds true not only for the general social and political repercussions of a political statement, but also for early neurophysiological responses in the brain of the hearer. Specifically, we show that the speaker’s perceived ability to bring about the state of affairs described in the message (his/her “potency to act”) is instrumental in shaping the hearer’s initial neurophysiological reaction to a political message. This result calls for a fundamental revision of existing perspectives on the neural implementation of language.

Many current approaches to the neurocognition of language emphasize the rapidity with which the human brain utilizes language-external information sources in the computation of a linguistic message. Thus, as shown by Hagoort and colleagues [2], there is no timing difference in the neurophysiological response to impossible statements (e.g. “The Dutch trains are sour and very crowded”) in comparison to statements that describe a possible scenario which happens to be false in the real world (e.g. “The Dutch trains are white and very crowded”, when presented to Dutch students who know that trains in the Netherlands are typically yellow). Both types of statements engender a highly comparable electrophysiological reaction in comparison to plausible controls (e.g. “The Dutch trains are yellow and very crowded”), namely a negativity with a peak latency of approximately 400 ms post critical word onset: the well-known N400 [3]. (Specifically, N400 onset and peak latency were indistinguishable between the two effects, but N400 amplitude was somewhat more pronounced for impossible statements as opposed to word knowledge violations.) Modulations of the N400 were also demonstrated for incompatibilities between speaker and message (e.g. “Every evening I drink some wine before I go to sleep”, when spoken in a child’s voice [4]) and between the message and co-speech gestures [5]. These findings have been taken to suggest that the brain rapidly integrates all available information sources in the online decoding of meaning [6], [7]. However, while these previous studies provide compelling evidence for a direct influence of sentence-external and even language-external factors on the computation of linguistic meaning, they all measured brain responses to mismatches between the message being conveyed and another information source (e.g. world knowledge, speaker identity etc.) such that the message was rendered implausible by the additional information.

In addition to these language-external influences on the online computation of linguistic meaning, recent research suggests that language comprehension may be directly modulated by the listener or reader’s current perspective on the world [8]–[13]. This includes both personal convictions as well as one’s present emotional state (mood). For example, van Berkum and colleagues [13] presented participants with statements such as “I think euthanasia is an unacceptable/acceptable course of action” and observed a small N400 increase for statements that did not correspond to participants’ personal convictions. The N400 has also been shown to be modulated by mood congruence, i.e. N400 amplitudes were larger when participants’ present mood (happy, sad, neutral) did not match the outcome of a short discourse (happy or sad) [12] (for a similar result at the single word level, see [10]). From results such as this, Egidi and Nusbaum concluded that self-referential factors such as mood play a very similar role to the language-external factors described above: “mood can influence the integration process in discourse comprehension by creating constraints on what would be a fitting ending” ([12], p.400).

It has not yet been investigated to date, however, how social attributions to the speaker of a particular message affect early online comprehension – for example, how a change of speaker from President Reagan to an unknown American citizen would have influenced the neural processing of the classic “Tear down this wall” sentence. By contrast, the crucial role of social cognition for language has been demonstrated clearly in a range of other domains. For example, social factors are central to ontogenetic development as they play a key role in language learning [14]. The relationship between language and social mechanisms such as Theory of Mind (ToM) or mentalizing has also been stressed from the perspective of cognitive evolution [15]. Further converging support stems from the observation of overlapping neural networks for language and mentalizing tasks (e.g. in medial prefrontal regions, the posterior superior temporal sulcus/temporo-parietal junction, anterior temporal cortex and left inferior frontal gyrus), particularly when language is presented as a connected narrative ([16], [17], for reviews). There is also some evidence to suggest that the neural networks supporting ToM may be subject to modulation by linguistic factors (e.g. one’s native language; [18]). Nevertheless, the tightness of the neural link between language and ToM remains a controversial issue (for arguments in favor, see e.g. [19]–[21]; for arguments against, see [22], [23]). Some of these discrepancies could be reconciled by the assumption that language and social cognition interact only in certain respects, i.e. that “pragmatic aspects of language affect ToM more than constitutive aspects [e.g. syntax and semantics]” [18]. In this view, language and social cognition are implemented in separable brain systems which do not rely on one another, but may ultimately interact in the realization of communicative goals. This perspective is again compatible with the assumption that language-related neural activity is primarily orchestrated by the computation of linguistic meaning.

Here, we use electrophysiology to test the stronger hypothesis that aspects of social cognition impact directly upon the computation of linguistic meaning during real-time language processing in the human brain. We will term this hypothesis the Linguistic Social Threshold (LST) hypothesis: the assumption that real-time language understanding is mediated directly by the social relevance of the statement being uttered. Specifically, we propose that the social relevance of a statement depends on the degree to which the speaker has the ability to bring about the state of affairs described in his/her utterance: his/her “potency to act” upon his/her words. For example, the social relevance (for a citizen of the European Union) of the statement “Germany plans to leave the Euro-zone” would be considerably higher if it were to be uttered by the German chancellor in a television interview than if it were proclaimed by one’s next-door-neighbor.

This hypothesis was tested empirically in the present study by means of event-related brain potentials (ERPs). As language comprehension is a very rapid process, with each new incoming word integrated with the preceding discourse within several hundred milliseconds [24], [25], timing is of the essence if the directness of an influence is to be assessed. This renders ERPs, a direct measure of neural activity with a temporal resolution in the millisecond range, ideally suited to our purposes. We presented videotaped statements pertaining to general world knowledge and politics (current affairs) that were either plausible (true) or implausible (false) and that were spoken either by a top political decision maker (the German Federal Minister of Finance), a prominent media personality (a well-respected former German news anchor) or a control speaker whom participants could not identify. By means of this design, we tested the hypothesis that real-time language understanding is directly mediated by knowledge about the social status of the speaker and his/her potency to act in the domain described.

## Materials and Methods

We conducted two experiments using event-related brain potentials (ERPs). Both studies involved the presentation of videotaped single-sentence utterances which were either plausible (true) or implausible (false) and either related to general world knowledge or to current political states of affairs. In Experiment 1, participants were presented with videos showing the German Federal Minister of Finance (at the time at which the experiment took place), Peer Steinbrück (http://en.wikipedia.org/wiki/Peer_Steinbrueck, accessed on June 16 2013), and a control speaker who was not known to participants (a professor of Germanic Linguistics at the University of Marburg). During the time of data acquisition, Peer Steinbrück was consistently rated as the third-most popular federal politician in Germany. (The ratings were taken from representative opinion polls collected by the German television station ZDF: http://politbarometer.zdf.de, accessed on June 16 2013. For these polls, which are conducted at least once a month, approximately 1250 Germans who are eligible to vote are questioned by telephone.) Experiment 2 was designed as a control study to ensure that any differences observed between the finance minister and the control speaker in Experiment 1 could not be attributed simply to the difference between an identifiable, high-profile speaker and an unidentifiable control. To this end, it employed exactly the same design and procedure as Experiment 1, but replaced the videos of the finance minister with videos of Ulrich Wickert, a former news anchor and one of the best-known and most highly respected media personalities in Germany (cf. http://en.wikipedia.org/wiki/Ulrich_Wickert, accessed on June 16 2013). Note that we conducted two experiments rather than including both prominent speakers in one study, as a single study with three speakers would have required the recording of a higher number of sentences. This was not feasible in view of the time constraints imposed by the schedules of our two well-known speakers. Nevertheless, as all other factors besides the identity of the prominent speaker were held constant across the two studies, we directly compared the results of both experiments within a single analysis involving the between-participants factor GROUP.

### Ethics statement

The present study was performed in accordance with the ethical standards laid down in the Declaration of Helsinki. Participants gave written informed consent before the beginning of the experiment and were informed that they could discontinue the study at any time should they wish to do so. The experimental protocols were approved by the ethics committee of the Max Planck Institute for Human Cognitive and Brain Sciences.

### Participants

Eighteen native speakers of German participated in each of the two experiments after giving written informed consent (Experiment 1∶10 women, mean age 24.67, range 21–29 years; Experiment 2∶8 women, mean age 25.0, range 21–35). No participant took part in both studies. All participants were right-handed, had normal or corrected-to-normal vision and good auditory acuity. None of them reported neurological or psychological disorders. A pre-test ensured that all participants recognized the high-profile speaker in the experiment in which they were taking part and that they did not recognize the control speaker.

## Materials

The sentence materials comprised thirty-six sets of the four conditions in Table 1. Implausible (false) and plausible (true) sentences for a particular statement type (general, political) were constructed as minimal pairs, ensuring that critical words were identical across conditions. The critical word always occurred sentence-finally. Sentence complexity (and length) was balanced across conditions for each statement type by ensuring that, for each true sentence, there was a structurally similar false sentence and vice versa.

**Table 1:** Example sentences for each of the four critical conditions in the present study.

Condition		Example
General	true	Michael Jackson ist ein Popsänger.+ *
		*Michael Jackson is a pop singer*
		Urlaub dient der Erholung des Arbeiters.
		*Vacations allow for the recuperation of the worker.*
	false	Fidel Castro ist ein Popsänger.*
		*Fidel Castro is a pop singer*
		Überstunden dienen der Erholung des Arbeiters.
		*Vacations allow for the recuperation of the worker.*
Political	true	Die Bundeskanzlerin plädiert für einen späteren Beitritt der Ukraine in den NATO-Verbund.*
		*The chancellor advocates a later entry of the Ukraine into the NATO alliance.*
		Die Pressestelle des Bundestages verfolgt die täglichen Meldungen der Bild-Zeitung.
		*The press office of the German Federal Parliament follows the daily reports of the Bild newspaper.* ( = the largest German tabloid newspaper)
	false	Die Bundesregierung verkündet den Austritt aus dem NATO-Verbund.*
		*The federal government announces the withdrawal from the NATO alliance.*
		Das Finanzministerium verteidigt die Subventionierung der Bild-Zeitung.
		*The Ministry of Finance defends the subsidization of the Bild newspaper.* ( = the largest German tabloid newspaper)

Critical words are underlined. Example videos showing each of the three speakers and further sentence examples for each condition are provided in the Supporting Information.

+ Note that this sentence was true/plausible at the time at which the experiment was conducted.

* Video examples for this sentence are provided in the supplementary materials.

Materials were selected on the basis of a questionnaire pre-test, in which forty native speakers of German rated sentences for their plausibility on a 6-point scale (1 = “highly plausible”, 6 = “completely implausible”). None of the participants in the pre-test also took part in the subsequent ERP studies. This ensured that, in the materials selected for inclusion in the ERP study, the plausible sentences were reliably rated as more plausible than their implausible counterparts (see the Supporting Information, Table S1, for rating results).

The 144 critical sentences selected for inclusion in the ERP studies (36 per condition) and 72 additional filler sentences were spoken by three different speakers: the German Federal Minister of Finance at the time of the experiment (Peer Steinbrück), a well-known and highly respected media personality (Ulrich Wickert) and a control speaker who was unknown to the participants of the experiment (a professor of Linguistics at the University of Marburg). Speakers were filmed by a professional camera team as they read the sentences from a teleprompter in a randomized order. The audiovisual setting (e.g. lighting, sound, background, clothing worn by the speaker) was comparable across the three recording sessions. Sentences containing errors, disfluencies, atypical prosodic contours or other problems were re-recorded at the end of the session. For presentation in the ERP study, the film material was cut into segments containing single sentences and compressed (MPEG4-format). See the supplementary videos (Videos S1–S12) for examples.

In Experiment 1 (minister of finance), we presented videos of Peer Steinbrück and the control speaker. To this end, the 288 critical videos (36 per condition and speaker) were subdivided into two lists of 144 videos each (18 per condition and speaker) and interspersed with 72 filler videos (36 of each speaker). The two lists, each containing 216 videos in total, were presented to participants in different pseudo-randomized orders. List presentation was counterbalanced across participants. Each participant saw a single list once.

Materials for Experiment 2 (media personality) were prepared in the same way as for Experiment 1, with the exception that the videos of Peer Steinbrück were replaced with those of Ulrich Wickert.

### Procedure

**Figure 1:**
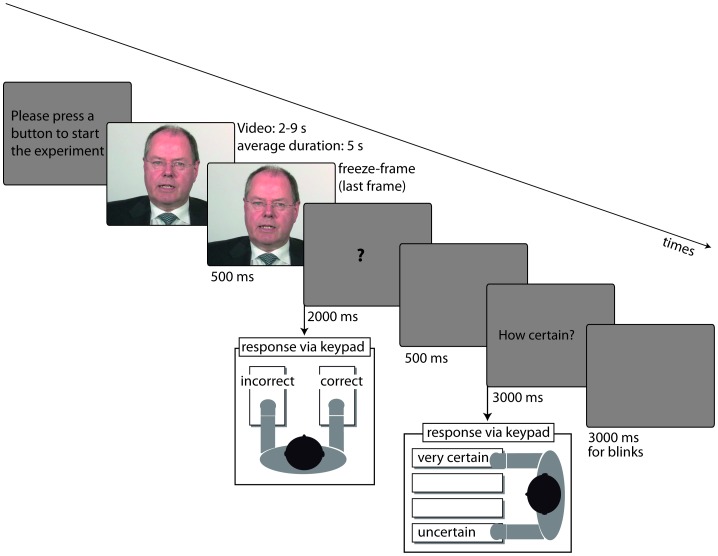
Schematic illustration of the experimental procedure in the present study (Experiments 1 and 2). The speaker shown gave written informed consent to the publication of his photo.

The experimental procedure (cf. Figure 1) was identical for Experiments 1 and 2. Experimental sessions were conducted in a dimly lit, sound attenuated room. Participants sat in a comfortable chair, approximately 1 m in front of a 19 inch computer screen. Videos were presented in a full-screen mode and ended with a 500 ms freeze-frame. Subsequently, participants judged whether the preceding statement was true or false by pressing one of two buttons on a dual game controller (maximal reaction time: 2000 ms). After a further 500 ms of blank screen, participants were required to indicate how certain they were of their answer (4-point scale; maximal reaction time: 3000 ms). Following an inter-trial interval of 3000 ms, the next trial started. Participants were asked to avoid movements and eye-blinks during the presentation of the videos.

Experimental sessions began with a short training session followed by 4 experimental blocks of 54 videos each, between which the participants took short breaks. Finally, participants completed a debriefing questionnaire in which they were asked about their impression of the speakers that they had just viewed. Specifically, speakers were rated on dimensions known to influence peoples’ impressions of political leaders (“warmth” and “competence:”) [26]. We used judgements of how likable participants judged the speaker and to what degree they judged him able to assert himself as measures of the warmth and competence dimensions, respectively. Each experimental session lasted approximately 2 hours, of which the ERP experiment itself comprised approximately 40 min.

### EEG recording and preprocessing

EEG recording and preprocessing was identical for Experiments 1 and 2. The EEG was recorded by means of 27 electrodes fixed at the surface of the scalp by means of an elastic cap (Easycap GmbH, Herrsching-Breitbrunn, Germany). EEG-signals were recorded from 27 electrodes positioned according to the international 10–20 system (ground: AFz, reference: right mastoid; offline re-referencing to linked mastoids). The electrooculogram (EOG) was monitored by means of electrodes placed at the outer canthus of each eye (horizontal EOG) and above and below the participant’s right eye (vertical EOG). Impedances were kept below 5 kOhm. Channels were amplified using a BrainVision BrainAmp amplifier (Brain Products GmbH, Gilching, Germany; digitization rate: 500 Hz). Raw EEG data were filtered offline (0.3–20 Hz bandpass). Individual participant EEGs were scanned automatically and manually for artifacts (blinks, other EOG artifacts, movement-based artifacts etc.); the automatic EOG rejection criterion was 40 µV. Trials containing artifacts were excluded from further analysis.

For the ERP plots, average ERPs per participant, condition and electrode were calculated from −200 to 1000 ms relative to the critical sentence-final word (artifact-free trials only) before grand-average ERPs were calculated over all participants. For the statistical analysis, which included both by-participant and by-item variance, average ERP amplitudes were calculated by participant, item, electrode, condition and time window and entered into a linear mixed model analysis (see below).

### Statistical analyses

For both rating tasks, mean error rates (true-false judgment) and certainty rates (certainty judgment) per condition were analyzed using linear mixed effects models in R [27] and the lme4 package [28] with participants and items as crossed random effects [29]. The fixed factors included in the analysis were: SENTENCE-TYPE (political versus general statements), TRUE-FALSE (statement is true versus false), SPEAKER (public figure versus unidentifiable control speaker) and GROUP (Experiment 1– prominent speaker: finance minister versus Experiment 2– prominent speaker: media personality). Following [30], we calculated models with maximal random effects structures to ensure maximal generalizability. Thus, in addition to including random intercepts by participant and item, models included by-participant random slopes for SENTENCE-TYPE, TRUE-FALSE and SPEAKER and by-item random slopes for SPEAKER and GROUP. For the fixed factor structure of each model, we used an iterative model simplification procedure to determine the minimally adequate model (i.e. non-significant effects were removed from the model as long as this did not decrease overall goodness of model fit) [31].

For the ERP data, mean amplitude values per participant, condition and time window of interest were analyzed by means of linear mixed effects models. In addition to the fixed effects included in the analysis of the behavioral data, the ERP data analysis included the topographical factor region of interest (ROI). ROIs were defined as follows: left-anterior (F7, FC5, FC1), left-central (T7, C3, CP5), left-posterior (P7, P3, O1), right-anterior (F8, FC5, FC2), right-central (T8, C4, CP6), and right-posterior (P8, P4, O2). As in the analysis of the behavioral data, models were fit using maximal random effects structures, i.e. by-participant random slopes for SENTENCE-TYPE, TRUE-FALSE, SPEAKER and ROI and by-item random slopes for SPEAKER and GROUP as well as random intercepts by participant and item. For fixed factors, model fitting proceeded as described above for the behavioral data. As p-values cannot currently be estimated for mixed effects models with random correlation parameters, we follow Baayen [32] in treating fixed effects with an absolute t-value >2 as significant. In the following, we report only those effects approaching significance (|*t*| >1.9).

## Results

### Behavioral responses

**Figure 2:**
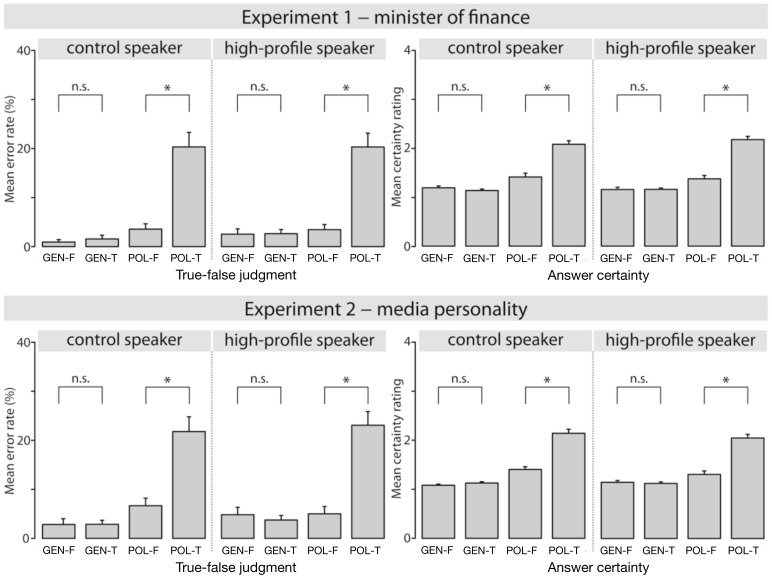
Results of the behavioral tasks. Results for Experiment 1 are shown in the top panel and results for Experiment 2 are shown in the bottom panel. In both cases, mean error rates for the true-false judgment are shown on the left-hand side, whereas mean certainty judgments are shown on the right-hand side. For the certainty judgments, the four-point scale was defined as follows: 1 (very certain) –4 (completely uncertain).

Participants’ behavioral responses (mean error rates for the true-false judgment task and mean certainty ratings) are depicted in Figure 2. As is apparent from the figure, both experiments showed very similar data patterns for both tasks and neither experiment revealed any influences of the factor SPEAKER.

The statistical analysis confirmed these descriptive impressions. For the true-false judgment task, a logit linear mixed effects model revealed an interaction of SENTENCE-TYPE × TRUE-FALSE (Estimate: 1.72, Standard error: 0.54, *z* = 3.19, *p*<0.01). This interaction was due to higher error rates for true versus false political statements (separate model for political statements, effect of TRUE-FALSE: Estimate: 2.02, Standard error: 0.43, *z* = 4.70, *p*<0.0001), while there was no significant difference for general knowledge statements (*p*>0.5).

The mixed model analysis of the certainty ratings showed a very similar result, namely an interaction of SENTENCE-TYPE × TRUE-FALSE (Estimate: 0.74, Standard error: 0.09, t = 7.91), which was due to lower certainty ratings for true versus false political statements (Estimate: 0.74, Standard error: 0.10, t = 7.63), while there was no difference for general knowledge statements (t <1).

Overall, in spite of the higher error rates and lower certainty ratings for true political statements as opposed to other sentence types, the high accuracy and certainty for judgments of false political statements shows that participants had no problems in identifying implausible political utterances. In addition, as the behavioral data showed no effects of speaker, they cannot account for our ERP findings (see below).

### Event-related brain potentials

**Figure 3:**
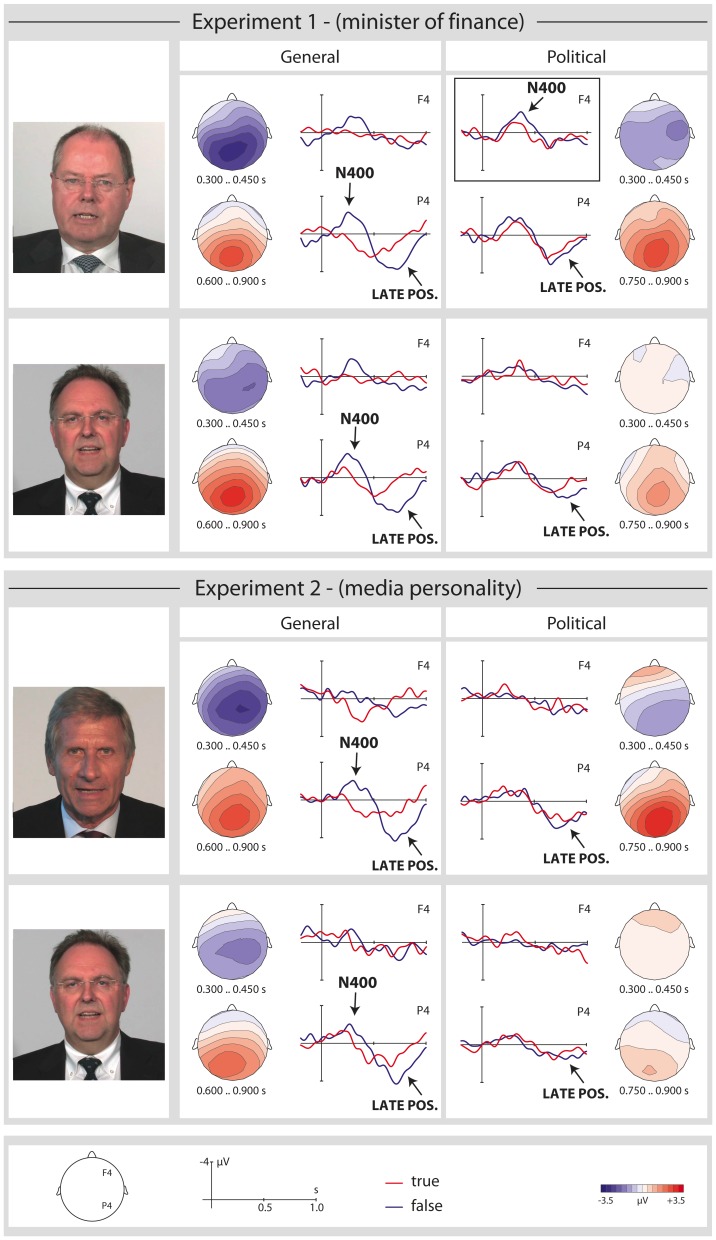
Grand average event-related brain potentials (ERPs) timelocked to the critical word (onset at the vertical bar) for general and political statements in Experiment 1 (top panel) and Experiment 2 (bottom panel). For each experiment, the higher panel shows the ERP responses for the high-profile speaker whereas the lower panel shows the ERP responses for the control speaker. ERPs are depicted for two selected electrodes, while the distribution of the N400 and late positivity effects is shown by the topographical maps (false – true). The frame in the top right-hand corner highlights the selective electrophysiological response for false political statements made by the finance minister. Negativity is plotted upwards. Speakers gave written informed consent to the publication of their photos in this figure.

Grand average ERPs for true and false general and political statements are shown in Figure 3. For statements pertaining to general knowledge (Figure 3, left panel) false versus true sentences showed a broadly distributed negativity (N400) [3], [33], [34] between approximately 150 and 450 ms post critical word onset. Furthermore, false statements engendered a late parietal positivity (P600) [35] between 600 and 900 ms in comparison to their true counterparts. Both of these effects appear very similar for both Experiments and across speakers. ERPs to political statements (Figure 3, right panel), by contrast, revealed a striking difference between speakers: an N400-like negativity for false statements was only apparent for the high profile political speaker (the Minister of Finance in Experiment 1), but not for the unidentifiable control speaker in Experiment 1 nor for either speaker in Experiment 2. A small late positivity for plausible vs. implausible statements was apparent for all speakers in both experiments. The ERP results were analyzed statistically in four time windows: 150–300 ms and 300–450 ms for the N400 and 600–750 ms and 750–900 ms for the late positivity. Two time windows were selected for each effect as visual inspection suggested that onset latencies might differ between different statement types (i.e. effects appeared to be somewhat earlier for general as opposed to political statements). By means of two time windows per effect, we aimed to test whether this descriptive impression was indeed borne out statistically (for the use of multiple time windows to assess quantitative differences in language-related ERP effects, see for example [36]). Additional control analyses between 0 and 150 ms did not reveal any significant effects.

#### N400 time window 1 (150–300 ms)

The minimal adequate model for the 150–300 ms time window (see Table S2 for the full model specification) showed an interaction of GROUP × SPEAKER × TYPE × TRUE-FALSE. We thus fit separate models for each statement type (general and political). Both political and general statements showed an interaction of GROUP × SPEAKER × TRUE-FALSE (political: Estimate: −0.36, Standard error: 0.16, t = −2.29; general: Estimate: 1.54, Standard error: 0.41, t = 3.76). The model for general statements additionally revealed a significant interaction of GROUP × SPEAKER × TRUE-FALSE × ROI (|*t*s| >2 for the left-posterior, right-central and right-posterior ROIs).

Further separate model fits per GROUP showed an interaction of SPEAKER × TRUE-FALSE × ROI for political statements in Experiment 1 (Minister of Finance; |*t*s| >2 for the left-posterior and right-posterior ROIs), but no comparable effect in Experiment 2 (media personality; all |*t*s| <1.1 for SPEAKER × TRUE-FALSE × ROI, |*t*| <1 for SPEAKER × TRUE-FALSE). We followed up on the interaction for political statements in Experiment 1 by conducting paired t-tests for true versus false statements per speaker and ROI. These revealed significant effects of TRUE-FALSE for the political speaker in both posterior ROIs and the right-central ROI (all |*t*s| >2.1, all *p*s <0.5). (For the control speaker, a significant effect of TRUE-FALSE emerged in the right-central region (t(17) = −2.66, p<0.02). However, this effect was reversed in polarity to the general N400 effect observed for false versus true statements, i.e. ERPs to false statements were more positive-going than ERPs to true statements.).

For the general statements, separate model fits per GROUP showed interactions of SPEAKER × TRUE-FALSE × ROI for both experiments (Experiment 1, Minister of Finance: |*t*s| >2 for the left-posterior and right-posterior ROIs; Experiment 2, media personality: |*t*| >2 for the right-posterior ROI). For Experiment 1, paired t-tests revealed significant effects of TRUE-FALSE for the political speaker in both central and both posterior ROIs (all |*t*s| >2.1, all *p*s <0.5) and for the control speaker in all left-hemispheric ROIs and the right-posterior ROI (all |*t*s| >2.1, all *p*s <0.5). For Experiment 2, paired t-tests reached significance in all regions for the media personality (all |*t*s| >2.3, all *p*s <0.03), but there were no significant effects for the control speaker (all |*t*s| <1.2, all *p*s >0.2).

In summary, the analysis of the early N400 time window showed a centro-parietal negativity for false versus true political statements only for the Minister of Finance, but not for either of the other two speakers. For general knowledge statements, we observed a broadly distributed negativity for false versus true sentences for both prominent speakers. For the control speaker, by contrast, effects for general knowledge statements were unreliable in this time window: an effect of TRUE-FALSE emerged in Experiment 1, but not in Experiment 2.

#### N400 time window 2 (300–450 ms)

In the second N400 time window, the minimal adequate model (see Table S3) again revealed an interaction of GROUP × SPEAKER × TYPE × TRUE-FALSE. Separate models per statement type showed an interaction of GROUP × SPEAKER × TRUE-FALSE for political statements (Estimate: 0.62, Standard error: 0.16, t = −3.90). The model for general knowledge statements, by contrast, only revealed an interaction of SPEAKER × TRUE-FALSE × ROI (both posterior ROIs: |*t*s| >2.0) but no additional interaction with GROUP.

Further analyses for political statements showed a marginally significant interaction of SPEAKER × TRUE-FALSE × ROI for Experiment 1 (Minister of Finance; Estimate: −0.74, Standard error: 0.39, t = −1.90) but no effects of TRUE-FALSE or interactions of SPEAKER × TRUE-FALSE were observable for Experiment 2 (media personality; all |*t*s| <1). We followed up on the SPEAKER × TRUE-FALSE × ROI interaction in Experiment 1 with t-tests per ROI and speaker. These revealed significant effects of TRUE-FALSE for the political speaker in anterior and central regions (all |*t*s| >2.5, all *p*s <0.03), but no effects for the control speaker in any ROI (all |*t*s| <1.1, all *p*s >0.3).

For the general knowledge statements, we followed up on the interaction of SPEAKER × TRUE-FALSE × ROI by conducting t-tests per speaker and ROI, collapsing over experiments (since there was no indication of a GROUP-based modulation of the TRUE-FALSE effect). These pairwise comparisons revealed significant effects of TRUE-FALSE in each ROI for both levels of the factor SPEAKER (i.e. for the prominent speakers and the control speaker). The interaction was due to the fact that the effects were more pronounced for the prominent speakers (all |*t*s| >4.6, all *p*s <0.0001) than for the control speaker (all |*t*s| >2.5, all *p*s <0.02).

To summarize the results of the second N400 time window, we again observed an N400-like negativity for false versus true political statements only for the Minister of Finance in Experiment 1 but not for either of the other speakers. False versus true general knowledge statements, by contrast, engendered an N400 effect for all three speakers, but this effect was more pronounced for the two prominent speakers (the Minister of Finance and the media personality) than for the unrecognizable control speaker.

#### Late positivity time window 1 (600–750 ms)

The analysis of the first late positivity time window (Table S4) showed an interaction of TRUE-FALSE × TYPE × ROI, which was explored further by means of separate models for each sentence type. For political statements, neither the main effect of TRUE-FALSE nor the interaction TRUE-FALSE × ROI reached significance. For general knowledge statements, there was a significant interaction of TRUE-FALSE × ROI (|*t*s| >4.4 in central and posterior ROIs). T-tests per ROI revealed significant effects of TRUE-FALSE in each region (all |*t*s| >2.9, *p*s <0.01; t-values increasing from anterior to central to posterior).

In summary, the first late positivity time window showed an increased positivity effect for false versus true general knowledge statements, which did not differ across speakers. By contrast no positivity effects were observable for political statements.

#### Late positivity time window 2 (750–900 ms)

The global model for the second late positivity window (Table S5) did not converge with a maximal random effects structure. Thus, the random effects structure was simplified by removing the by-participants random intercept for speaker, which was the random intercept with the smallest variance (following the suggestions for random effects simplification in [30]). The simplified model showed a marginally significant interaction of GROUP × SPEAKER × TYPE × TRUE-FALSE × ROI and a significant interaction of GROUP × SPEAKER × TYPE × TRUE-FALSE. Separate models for the two statement types showed interactions of GROUP × SPEAKER × TRUE-FALSE × ROI in both cases (political: t >2.0 in the left-central ROI; general: t >2.2 in the left-posterior ROI).

The source of these interactions was examined by means of separate models per statement type and experiment. For political statements, Experiment 1 (Minister of Finance) showed a marginally significant interaction TRUE-FALSE × SPEAKER × ROI (|*t*| = 1.90 in the right-posterior ROI). Follow-up paired t-tests per SPEAKER and ROI revealed significant effects of TRUE-FALSE for the political speaker in both central regions and the left-posterior region (all |*t*s| >2.1, *p*s <0.05) and for the control speaker in both posterior regions and the left-central region (all |*t*s| >2.4, *p*s <0.03). Thus, the positivity had very slightly differing distributions for the two speakers. For Experiment 2 (media personality), the analysis of the political statements only revealed an interaction of TRUE-FALSE × ROI (|*t*| >2.7 in the left-central ROI) but no interactions with SPEAKER. Follow-up t-tests per ROI showed a significant effect of TRUE-FALSE in the left-central ROI (t(17) = 3.13, p<0.01).

For general knowledge statements, the model for Experiment 1 (Minister of Finance) showed an interaction of TRUE-FALSE × ROI (|*t*s| >3.5 in central and posterior ROIs), which was due to significant effects of TRUE-FALSE in all regions except the left-anterior ROI (all |*t*s| >2.7, *p*s <0.02). In Experiment 2 (media personality), the interaction TRUE-FALSE × SPEAKER × ROI reached significance (|*t*s| >2.5 in both posterior ROIs). Separate t-tests per SPEAKER and ROI showed significant effects of TRUE-FALSE in all regions for the former news anchor (all |*t*s| >3.0, *p*s <0.01), but only in central and posterior ROIs for the control speaker (all |*t*s| >2.7, *p*s <0.02).

In summary, the analysis of the second positivity time window showed positivities for false versus true statements of both types and for all three speakers, though with slightly differing distributions. The positivity for political statements was not very pronounced in Experiment 2 (media personality), reaching significance in only the left-central region. In Experiment 1 (Minister of Finance), by contrast, it was observable in central and posterior regions. The positivity for general statements was generally more widely distributed, reaching significance in all regions but the left-anterior ROI in Experiment 1, in all ROIs for the media personality in Experiment 2 and in central and posterior ROIs for the control speaker in Experiment 2.

### Correlations between ERP responses and participants’ subjective evaluation of the political speaker

While the findings presented above show a direct influence of the speaker’s social status and the content of the message being conveyed on neurophysiological reactions, we additionally conducted a more direct test of the hypothesis that this effect was indeed a function of participants’ subjective evaluation of the speaker. To this end, we correlated the ERP effect observed for false versus true political statements spoken by the finance minister in Experiment 1 with participants’ ratings of speaker-related characteristics. Specifically, we sought to examine whether this effect, which was specific to the political statements uttered by a politician, correlated with the warmth and competence dimensions, which have been shown to influence peoples’ impressions of political leaders. To address each of these two dimensions, we correlated the amplitude of the “political ERP effect” with participants’ ratings. The two rating dimensions (z-normalized values of the original 7-point scale ratings) and their interaction were entered as predictor variables into a linear mixed effects model with the difference of mean ERP amplitudes (false political - true political statements) per participant and ROI in the 300–450 ms time window in Experiment 1 (in which the effect was most pronounced) serving as the dependent variable. Model parameters are summarized in Table 2.

**Table 2:** Parameter values for fixed effects in the best-fitting linear mixed-effects model of mean amplitude differences for the political sentences (false - true) in the 300–450 ms time window for the political speaker in Experiment 1.

Effect	Estimate	Standard error	*t-*value
Intercept	−0.98	0.21	−4.61
ASSERTIVE*LIKABLE	−0.84	0.23	−3.58
LIKABLE*ROI(right-posterior)	−0.66	0.33	−2.03
ASSERTIVE*LIKABLE*ROI(left-posterior)	0.72	0.35	2.07
ASSERTIVE*LIKABLE*ROI(right-central)	0.60	0.26	2.30
ASSERTIVE*LIKABLE*ROI(right-posterior)	1.29	0.36	3.62

In addition to the fixed effects Assertiveness, Likability, ROI and their interactions, the model included random intercepts by participant and by-participant random slopes for ROI.

Abbreviations: LIKABLE - how likable individual participants judged the political speaker to be (indicative of the warmth dimension); ASSERTIVE - how likely they judged him to be able to assert himself (indicative of the competence dimension).

**Figure 4:**
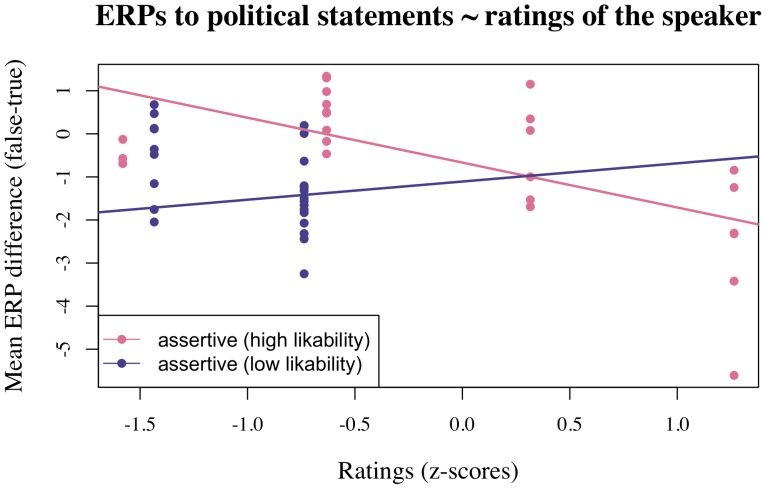
Effect of by-participant assertiveness ratings for the political speaker on the TRUE-FALSE effect for political statements in the left-anterior region in Experiment 1. Higher N400 amplitudes correlated with higher assertiveness judgements for those participants who judged the political speaker to be of above-median likability.

**Figure 5:**
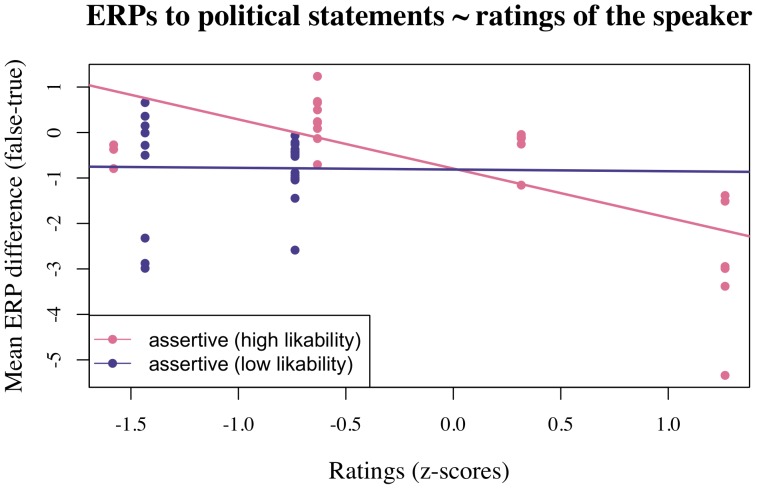
Effect of by-participant assertiveness ratings for the political speaker on the TRUE-FALSE effect for political statements in the right-anterior region in Experiment 1. Higher N400 amplitudes correlated with higher assertiveness judgements for those participants who judged the political speaker to be of above-median likability.

As is apparent from Table 2, mixed effects modeling revealed that assertiveness and likability interacted to predict ERP amplitudes for the political plausibility effect for the finance minister in Experiment 1 and that this interaction was modulated by ROI. Additional analyses per ROI revealed interactions of assertiveness and likability in both anterior regions (left: Estimate: −0.84 (CI: −1.20– −0.49), p<0.001; right: Estimate: −0.61 (CI: −0.95– −0.26), p<0.02). To further examine the nature of these interactions we conducted a median split on likability judgements and modeled the effect of assertiveness separately for those participants who judged the political speaker to be of higher-than-median and lower-than-median likability, respectively, in each of the two anterior ROIs (see Table 3 and Figures 4 and 5). This analysis revealed that N400 amplitude for the political sentences correlated significantly with assertiveness judgements only for the participants who judged the political speaker to be of higher-than-median likability. Specifically, in this group, higher assertiveness ratings correlated with larger N400 amplitudes (i.e. more negative N400 effects for implausible vs. plausible political statements). These findings thus demonstrate the modulation of the N400 effect observed for political statements spoken by the finance minister were dependent on participants’ subjective rating of the speaker along personality-related dimensions (warmth and competence) that are also known to influence impressions of political leaders. Crucially, as visualized in supplementary Figure S1, a corresponding analysis of the general knowledge statements did not reveal any correlations between the N400 effect for false versus true statements and assertiveness or likability ratings. (Note that, while the global model did show an interaction of Assertiveness × ROI(right-central), Estimate: −0.71, Standard error: 0.34, t = −2.11, separate analyses per ROI did not reveal a significant effect of Assertiveness in any region.).

**Table 3:** Resolution of the Assertiveness × Likability interaction in the 300–450 ms time window for the anterior ROIs in Experiment 1.

Effect ofAssertiveness		Estimate	95% CI-lower	95% CI-upper	*p*-value
Left-anterior	low Likability	0.42	0.03	0.79	<0.06
	high Likability	−1.04	−1.72	−0.36	<0.04
Right-anterior	low Likability	−0.04	−0.46	0.39	>0.90
	high Likability	−1.08	−1.67	−0.49	<0.02

Effects of by-participant Assertiveness judgements on mean ERP amplitude differences in the Low-Likability (i.e. lower-than-median likability of the political speaker) and High-Likability (i.e. higher-than-median likability of the political speaker) groups as well as associated confidence intervals and p-values. Note that, since the by-ROI analyses reported here did not involve any random slopes, confidence intervals and p-values could be estimated using MCMC sampling [29].

## Discussion

The present findings confirm the Linguistic Social Threshold (LST) hypothesis by demonstrating that social aspects of the speaker-hearer relationship modulate the earliest brain response that indexes sentence-level meaning: the N400. We observed an increased N400 (between approximately 150 and 450 ms post critical word onset) for false versus true political statements only when they were spoken by a top political decision-maker, but not when the same statements were uttered by a well-known media personality or by a control speaker. By contrast, N400 effects were observable for all three speakers (politician, media personality, unidentifiable control speaker) for false versus true general knowledge statements. However, they were generally more pronounced for the high prominence speakers as opposed to the control speaker. Finally, a later electrophysiological response (late positivity) was observed for all false utterances irrespective of speaker and type of statement. In the following, we will discuss each of these effects in turn before describing the broader consequences of our findings.

### N400 effect for political statements

Crucially, the early speaker-based effect for political statements cannot be explained by speaker-induced changes in plausibility (as in previous studies [4], [37]), since participants’ ratings did not change as a function of the speaker. The effect was predicted, however, by participants’ subjective ratings of two personality-related characteristics of the political speaker, namely how assertive and likable they considered him to be. Note that it is possible that differences between speakers may have emerged with a different task, e.g. if participants were asked to judge how plausible they considered it to be for the speaker to make this particular statement. However, in contrast to previous studies examining the combination of speaker and message [4], [37], the political statements used here were false for all three speakers and participants had no difficulty in classifying them as such (cf. the low error rates for false political statements in Figure 2).

The modulation of the N400 via participants’ subjective personality impressions of the political speaker provides strong converging support for a socially-mediated interpretation of this effect. Strikingly, on an individual participant level, we observed larger N400 amplitudes for false vs. true political statements with increasing likability and assertiveness ratings of the political speaker. These two rating scales relate to two dimensions of social cognition which have been claimed as universals of how we judge others’ personality traits and which are termed “warmth” and “competence”, respectively by Fiske and colleagues (e.g. [26]): According to recent theory and research in social cognition, the warmth dimension captures traits that are related to perceived intent, including friendliness, helpfulness, sincerity, trustworthiness and morality, whereas the competence dimension reflects traits that are related to perceived ability, including intelligence, skill, creativity and efficacy”([26], p.770). The warmth and competence dimensions also manifest themselves in people’s evaluations of top-level politicians, both with regard to their spontaneous impressions of candidates for top political offices and with respect to their assessments of political leaders [38]–[40]. Strikingly, these dimensions also modulate the earliest neurophysiological response to sentence-level meaning when a hearer perceives a political statement uttered by a politician.

These findings thus provide a compelling demonstration that the neural computation of a linguistic message is rapidly influenced by the social status of the speaker. Importantly, the effect cannot simply be reduced to a difference between well-known (identifiable, prominent) speakers and an unidentifiable control speaker, since it was specific to the minister of finance and not observable for the media personality. This indicates that the combination of speaker and message content was crucial: the N400 effect for political statements was only observable for a top political decision-maker, but not for either of the other two speakers, irrespective of their degree of prominence.

We suggest that the key factor involved in engendering the political N400 effect may be characterized in terms of the potency of the speaker to act on the content encoded in the message: of the three speakers in the present study, only the Federal Minister of Finance – as a member of the governing cabinet – had the principled capacity to bring about the state of affairs described by a political statement. Thus, only this particular combination of speaker and message content was associated with possible real-world consequences for the listener. This conclusion is supported by the fact that general knowledge statements, which none of our speakers had the power to change, engendered N400 effects irrespective of the speaker. Our results therefore demonstrate that the neural mechanisms of utterance interpretation not only involve a fast and multi-modal evaluation of the likelihood of a statement in the present communicative context, but also a socially-oriented evaluation of the potential consequences that the combination of speaker and message might have for the listener.

### N400 effect for general knowledge statements

As already noted in the preceding subsection, general knowledge statements did not show a comparable speaker-based modulation of neurophysiological responses to political statements: here, false versus true statements elicited an N400 for all three speakers (though, as discussed in more detail below, the onset of this effect was delayed for the control speaker in Experiment 2). On the one hand, this observation lends support to the proposal advanced above, namely that the neural response to an utterance is crucially defined by the speaker’s potency to bring about the state of affairs described in his/her statement. This clearly does not apply to the general knowledge statements for any of our three speakers. On the other hand, however, the results for the general knowledge statements also show that speaker status as a general factor (i.e. whether a speaker is a well-known, public-domain personality or not) cannot be discounted completely in the interpretation of our results. We observed an interaction between speaker and plausibility for general knowledge statements, which was attributable to a more pronounced N400 effect for the prominent as opposed to the unidentifiable speaker. This difference manifested itself in terms of both amplitude (i.e. effects were generally more pronounced for the well-known speakers in the later N400 time window) and latency (i.e. the negativity effect for the control speaker did not reach significance in the earlier N400 time window in Experiment 2, when the prominent speaker was the media personality).

A possible explanation for the amplitude modulation is that the identifiability of the speaker leads to stronger expectations with regard to upcoming words within a sentence context. In other words: in an experimental context in which speakers are uttering both plausible and implausible statements, the prediction for a plausible sentence-completing word is strengthened when the speaker is identifiable as a public figure as opposed to when he/she is unidentifiable. For the well-known speakers, hearers are familiar with them from the media and are accustomed to them uttering (more or less) plausible statements – certainly not nonsensical sentences such as the implausible conditions in the present study. The unidentifiable speaker, by contrast, was only introduced to participants in the context of the experiment. Their experience with this particular speaker was thus limited to the 50%-to−50% ratio of plausible to implausible utterances constituting our experimental materials. Indeed, previous studies suggest that intra-experimental experience with a speaker’s “communicative style” can influence electrophysiological responses related to language processing [41]. Thus, it is possible that the perceived, relative unreliability of the control speaker – which, in contrast to the identifiable speakers, participants could only gauge on the basis of their intra-experimental experience – led to an attenuation of the linguistically-based prediction for the plausible sentence continuation. In accordance with current assumptions about the N400, which state that N400 amplitude modulations primarily reflect the degree of preactivation of a critical word via the prior sentence or discourse context [33], [42], this lower degree of predictability may have led to a lower degree of lexical preactivation of the plausible critical word and, hence, to a reduced N400 effect for the control speaker.

While, as noted above, an explanation along these lines is in accordance with current accounts of the the N400, it suggests a novel modulating factor for the crucial mechanism of lexical preactivation. While existing proposals have focused on the role of linguistic factors in modulating lexical preactivation, the present findings indicate that familiarity with the speaker may also play an important role in determining the strength of a prediction and, hence, the degree of preactivation for a predicted word. This assumption not only accounts for the present results in predicting the less pronounced N400 effects for general statements for the control speaker as opposed to the two well-known (public figure) speakers, but also helps to link this result to the findings for political statements and to previous studies on the relationship between speaker and message. With regard to previous studies, the plausibility mismatches induced between speaker and message [4], [37] could be explained along similar lines. When hearing a child’s voice uttering a sentence beginning such as “Every evening I drink a glass of …”, the degree of lexical preactivation for “wine” will be considerably lower than when the same sentence is uttered by an adult’s voice.

For the political statements in the present study, one could argue that the speaker’s potency to act on the message being described (see above) may have played a crucial role in modulating lexical preactivation: political statements uttered by a top-level politician can essentially be seen as statements of intent, thus leading to a higher degree of expectation/preactivation with increasing warmth and competence ratings of the speaker as a political leader. Without the heightened social relevance of the political statements that resulted from them being uttered by a top political decision-maker, predictability of the critical words in the true political sentences was apparently too low to engender an N400 effect for false versus true statements. This is supported by the results of the pretest, which showed that the plausibility difference between true and false political statements was smaller than that between the true and false general knowledge statements. Possibly, this could be taken to indicate that expectation/preactivation is only one of the mechanisms at work here and that relevance of the statement to the hearer – as determined via the combination of speaker and message – should be taken into account as an independent influence on the N400 and the neural computation of linguistic meaning more generally. However, this assumption will clearly require further corroboration from future research.

With regard to the latency modulation for the control speaker depending on identity of the prominent speaker (i.e. the plausibility effect reached significance in the earlier N400 time window only in Experiment 1), we can only speculate at present. As effects of the experimental environment have been attested in a number of previous electrophysiological studies on language processing (e.g. [43]–[46], for a more general cognitive theory that can derive such effects, see [47]), it appears possible that the identity of the prominent speaker may have impacted upon the processing of statements uttered by the control speaker. In other words: encountering an unknown speaker in the context of a famous media personality may influence language processing differently than if that same person were encountered in the context of a well-known politician. Yet, however intriguing this possibility may be, this rather speculative proposal clearly requires further corroboration in future research. In this context, it also appears important to note that expectation-based effects such as those discussed above typically modulate N400 amplitude rather than N400 latency (e.g. [48]) such that a possible effect of one speaker upon another would manifest itself as a novel modulation of N400 latency.

### The late positivity

As the main focus of the present study was on the rapidity with which the speaker’s social status impacts upon the neural correlates of processing a linguistic message, the late positivity is not central to our main hypotheses. Nevertheless, we believe that a few remarks on this effect – which was observable for all false versus true stimuli irrespective of speaker and statement type (though with differing latencies between statement types) – are in order.

Late positivity (P600) effects in language processing were originally characterized as indexing (syntactic) reanalysis [35], the processing of syntactic violations [49] or syntactic processing demands more generally [50]. More recently, however, it has become clear that these characterizations in terms of specific linguistic processing demands (e.g. syntactic processing) are too narrow, since late positive ERP effects are also observable for certain types of semantic violations (for recent reviews, see [51], [52]), appear to depend on the strength of the violation [53] and manifest themselves for a variety of different violation types, including orthographic violations [54]. Findings such as these have led to several more general proposals with regard to the functional nature of late positive effects in language processing, for example that they reflect conflict monitoring [43], [52], [54], [55] or stimulus categorization vis-à-vis an experimental task [56].

The present findings are highly compatible with these domain-general explanations of language-related late positivity effects. Both accounts predict a close relationship between the perceived plausibility of a statement – in the context of a judgement task – and late positivities, since plausibility judgements are tied to conflict monitoring and to categorization of a sentence as plausible or implausible. Since judgements did not differ between speakers in the current experiment, neither theory would predict a speaker-based modulation of the late positivity. Indeed, this was just what we observed, thus lending support to accounts of late positivity effects in language which stress their relationship with participants’ subjective evaluation of the stimulus rather than some inherent dimension of stimulus processing.

The latency difference between the positivity effects for political and general statements (i.e. the fact that the positivity only reached significance in the second positivity time window) is also predicted by this approach, as the latency of the domain-general P3 component is known to co-vary with stimulus processing and response-selection time [57]. As is apparent from the pretest and the certainty judgment task, political statements were more difficult to categorize as true or false than general statements, thus leading to a longer onset latency for the late positivity.

### Consequences for the neurobiology of language

The finding of an immediate influence of the speaker’s social status to language-related neurophysiological responses in the hearer is a novel result. In particular, our results go beyond previous demonstrations of early brain response (i.e. N400) modulations to a potentially plausible utterance that is rendered implausible or less likely by some contextual influence (e.g. world knowledge, discourse context, knowledge of the speaker) [2], [4], [58]. Rather, as noted above, the present study demonstrates a speaker-related modulation of the N400 response to clearly implausible political statements when uttered by a politician.

The present findings are difficult to reconcile with the view that language can be reduced to an abstract system of arbitrary symbols. Viewing a politician making a political statement has an immediate – rather than a delayed – effect on the neural response to an implausible utterance. Hence, there is no evidence for a two-step interpretation process in which linguistic meaning is calculated first and the social consequences of this meaning are assessed in a second step. Rather, the speaker’s social status and its potential implications for the message being conveyed has a direct impact on the computation of linguistic meaning. While this result is, to some degree, predicted by Hagoort and colleagues’ “one-step” theory of language processing [6], [7], it goes beyond previous findings in demonstrating a social influence that cannot be reduced to plausibility. It also extends previous findings of self-referential influences on language understanding (e.g. effects of personal convictions, mood) [8]–[13], by showing the effects of social attributions to a third person speaker.

At the same time, however, it is not straightforwardly clear how our findings might be derived within current embodied theories of language comprehension. Statements were identical in terms of the actions which they described and their plausibility was not modulated by the choice of speaker (cf. Figure 3).To explain the selective modulation of the N400 for political statements by a politician, an embodied theory would need to assume that “acting as a politician” is accessible to embodied simulation. However, this would contradict the assumption that simulation requires one to know how an action feels [59]. Our results thus support the perspective that sensory-motor simulation cannot account for the full range of findings on the attribution of intentions and mental states to others [60], [61] and extends these caveats to language understanding. At the very least, they call for an elaboration of embodied theories to explain how simulation might carry over to perspectives (as implied by a certain profession and the responsibilities that come with it) with which we have no personal experience.

If, as we have suggested above, a speaker’s potency to act upon the statement being uttered indeed plays a crucial role in conditioning the hearer’s neural response to this statement, this suggests that sentence interpretation draws upon action-related representations which are not purely based on sensory-motor simulation. Somewhat similar suggestions have recently been put forward for the word level [62], [63]. Clearly, future research will need to test this hypothesis and flesh out how these representations can be defined more concretely. A promising avenue of investigation is suggested by the observation that sentence understanding across different languages is centered around the identification of the participant primarily responsible for the state of affairs described in the sentence, the “actor”, and that processing effort is lowest when there is little competition for the actor role [64]–[67]. The fact that we are particularly attuned to (linguistically encoded) actors during language processing may have a more deeply-rooted, evolutionary origin. As suggested by Leslie: “Agents are a class of objects possessing sets of causal properties that distinguish them from other physical objects” and “as a result of evolution, we have become adapted to track these sets of properties and to efficiently learn to interpret the behaviour of these objects in specific ways” ([68], p.122). Thus, tracking (potential) actors, i.e. those entities that appear suited to bringing about changes in the environment (e.g. warranting a fight-or-flight response), allows us to interpret the world around us and make predictions about upcoming events (see also [69]). Further converging evidence for this assumption stems from the finding that the human attention system appears attuned towards monitoring humans and non-human animals as opposed to other categories: on the basis of several change-detection studies, New et al. [70] argue for a “visual monitoring system equipped with ancestrally derived animal-specific selection criteria” which “appears well designed for solving an ancient adaptive problem: detecting the presence of human and non-human animals and monitoring them for changes in their state and location”.

In summary, our results indicate that actor-centered representations, which are socially-conditioned but not sensory-motor in nature, play a crucial role in the neurocognition of language. Hence, beyond abstract and embodied theories, a new class of linguistic models is required.

## Supporting Information

Figure S1 **Correlations between by-participant assertiveness ratings, by-participant likability ratings and the interaction of assertiveness x likability for the political speaker and the TRUE-FALSE effect for general statements (ERP amplitudes for false - true statements in the 300–450 ms time window).** In contrast to political statements (see the main text and Figures 4 and 5), N400 amplitudes for false versus true general statements did not show a correlation with the likability and assertiveness of the political speaker.
(TIFF)

Video S1 **Example video for a plausible general knowledge statement uttered by the control speaker.** Note that all videos are marked with the text “Video material recorded for a scientific study” in order to avoid the possibility of them being misunderstood as actual video recordings of the two high-profile speakers when available on-line. This text did not appear in the videos during the experimental sessions, i.e. participants were not informed until after the experiment that the recordings had been made specifically for the purposes of this study. Speakers gave written informed consent to the publication of the videos.
(MOV)

Video S2 **Example video for a plausible general knowledge statement uttered by the Minister of Finance (Peer Steinbrück).** Note that all videos are marked with the text “Video material recorded for a scientific study” in order to avoid the possibility of them being misunderstood as actual video recordings of the two high-profile speakers when available on-line. This text did not appear in the videos during the experimental sessions, i.e. participants were not informed until after the experiment that the recordings had been made specifically for the purposes of this study. Speakers gave written informed consent to the publication of the videos.
(MOV)

Video S3 **Example video for a plausible general knowledge statement uttered by the Media Personality (Ulrich Wickert).** Note that all videos are marked with the text “Video material recorded for a scientific study” in order to avoid the possibility of them being misunderstood as actual video recordings of the two high-profile speakers when available on-line. This text did not appear in the videos during the experimental sessions, i.e. participants were not informed until after the experiment that the recordings had been made specifically for the purposes of this study. Speakers gave written informed consent to the publication of the videos.
(MOV)

Video S4 **Example video for an implausible general knowledge statement uttered by the control speaker.** Note that all videos are marked with the text “Video material recorded for a scientific study” in order to avoid the possibility of them being misunderstood as actual video recordings of the two high-profile speakers when available on-line. This text did not appear in the videos during the experimental sessions, i.e. participants were not informed until after the experiment that the recordings had been made specifically for the purposes of this study. Speakers gave written informed consent to the publication of the videos.
(MOV)

Video S5 **Example video for an implausible general knowledge statement uttered by the Minister of Finance (Peer Steinbrück).** Note that all videos are marked with the text “Video material recorded for a scientific study” in order to avoid the possibility of them being misunderstood as actual video recordings of the two high-profile speakers when available on-line. This text *did not* appear in the videos during the experimental sessions, i.e. participants were not informed until after the experiment that the recordings had been made specifically for the purposes of this study. Speakers gave written informed consent to the publication of the videos.
(MOV)

Video S6 **Example video for an implausible general knowledge statement uttered by the Media Personality (Ulrich Wickert).** Note that all videos are marked with the text “Video material recorded for a scientific study” in order to avoid the possibility of them being misunderstood as actual video recordings of the two high-profile speakers when available on-line. This text *did not* appear in the videos during the experimental sessions, i.e. participants were not informed until after the experiment that the recordings had been made specifically for the purposes of this study. Speakers gave written informed consent to the publication of the videos.
(MOV)

Video S7 **Example video for a plausible political statement uttered by the control speaker.** Note that all videos are marked with the text “Video material recorded for a scientific study” in order to avoid the possibility of them being misunderstood as actual video recordings of the two high-profile speakers when available on-line. This text *did not* appear in the videos during the experimental sessions, i.e. participants were not informed until after the experiment that the recordings had been made specifically for the purposes of this study. Speakers gave written informed consent to the publication of the videos.
(MOV)

Video S8 **Example video for a plausible political statement uttered by the Minister of Finance (Peer Steinbrück).** Note that all videos are marked with the text “Video material recorded for a scientific study” in order to avoid the possibility of them being misunderstood as actual video recordings of the two high-profile speakers when available on-line. This text *did not* appear in the videos during the experimental sessions, i.e. participants were not informed until after the experiment that the recordings had been made specifically for the purposes of this study. Speakers gave written informed consent to the publication of the videos.
(MOV)

Video S9 **Example video for a plausible political statement uttered by the Media Personality (Ulrich Wickert).** Note that all videos are marked with the text “Video material recorded for a scientific study” in order to avoid the possibility of them being misunderstood as actual video recordings of the two high-profile speakers when available on-line. This text *did not* appear in the videos during the experimental sessions, i.e. participants were not informed until after the experiment that the recordings had been made specifically for the purposes of this study. Speakers gave written informed consent to the publication of the videos.
(MOV)

Video S10 **Example video for an implausible political statement uttered by the control speaker.** Note that all videos are marked with the text “Video material recorded for a scientific study” in order to avoid the possibility of them being misunderstood as actual video recordings of the two high-profile speakers when available on-line. This text *did not* appear in the videos during the experimental sessions, i.e. participants were not informed until after the experiment that the recordings had been made specifically for the purposes of this study. Speakers gave written informed consent to the publication of the videos.
(MOV)

Video S11 **Example video for an implausible political statement uttered by the Minister of Finance (Peer Steinbrück).** Note that all videos are marked with the text “Video material recorded for a scientific study” in order to avoid the possibility of them being misunderstood as actual video recordings of the two high-profile speakers when available on-line. This text *did not* appear in the videos during the experimental sessions, i.e. participants were not informed until after the experiment that the recordings had been made specifically for the purposes of this study. Speakers gave written informed consent to the publication of the videos.
(MOV)

Video S12 **Example video for an implausible political statement uttered by the Media Personality (Ulrich Wickert).** Note that all videos are marked with the text “Video material recorded for a scientific study” in order to avoid the possibility of them being misunderstood as actual video recordings of the two high-profile speakers when available on-line. This text *did not* appear in the videos during the experimental sessions, i.e. participants were not informed until after the experiment that the recordings had been made specifically for the purposes of this study. Speakers gave written informed consent to the publication of the videos.
(MOV)

Table S1 Mean plausibility ratings for the critical sentence materials as determined in a questionnaire pre-test (standard deviations are given in parentheses). Ratings were obtained on a 6-point scale (1 = “highly plausible”, 6 = “completely implausible”). Statistical analysis of the ratings via a repeated measures analysis of variance (ANOVA) revealed a main effect of TRUE-FALSE (F(1,39) = 1555.73, p<0.0001) and interaction of SENTENCE-TYPE × TRUE-FALSE (F(1,39) = 142.94, p<0.0001). (Note that this analysis was performed by-participants only, since items differed across sentence types.) Simple comparisons for each sentence type were performed by-participants (F_1_) and by-items (F_2_) and are reported in the table.
(PDF)

Table S2 Parameter values for the fixed effects in the linear mixed effects model for the first N400 time window (150–300 ms). The model was fit using a maximal random effects structure and a minimal adequate fixed effects structure (see the main text for details). For reasons of readability, only effects approaching significance (|*t*| >1.9) are reported. In addition, in view of the research questions pursued here, we only report effects of or interactions including TRUE-FALSE. Note that the reference levels for the fixed factors were as follows: TRUE-FALSE: false; SENTENCE-TYPE: general; SPEAKER: control; GROUP: Experiment 1; ROI: left-anterior.
(PDF)

Table S3 Parameter values for the fixed effects in the linear mixed effects model for the second N400 time window (300–450 ms). The model was fit using a maximal random effects structure and a minimal adequate fixed effects structure (see the main text for details). For reasons of readability, only effects approaching significance (|*t*| >1.9) are reported. In addition, in view of the research questions pursued here, we only report effects of or interactions including TRUE-FALSE. Note that the reference levels for the fixed factors were as follows: TRUE-FALSE: false; SENTENCE-TYPE: general; SPEAKER: control; GROUP: Experiment 1; ROI: left-anterior.
(PDF)

Table S4 Parameter values for the fixed effects in the linear mixed effects model for the first late positivity time window (600–750 ms). The model was fit using a maximal random effects structure and a minimal adequate fixed effects structure (see the main text for details). For reasons of readability, only effects approaching significance (|*t*| >1.9) are reported. In addition, in view of the research questions pursued here, we only report effects of or interactions including TRUE-FALSE. Note that the reference levels for the fixed factors were as follows: TRUE-FALSE: false; SENTENCE-TYPE: general; SPEAKER: control; GROUP: Experiment 1; ROI: left-anterior.
(PDF)

Table S5 Parameter values for the fixed effects in the linear mixed effects model for the second late positivity time window (750–900 ms). The model was fit using a maximal random effects structure and a minimal adequate fixed effects structure (see the main text for details). Since the maximal model did not converge, the random effects structure was simplified by removing the by-participant random slope for speaker, as this was the random slope with the smallest variance (as suggested by [30]). For reasons of readability, only effects approaching significance (|*t*| >1.9) are reported. In addition, in view of the research questions pursued here, we only report effects of or interactions including TRUE-FALSE. Note that the reference levels for the fixed factors were as follows: TRUE-FALSE: false; SENTENCE-TYPE: general; SPEAKER: control; GROUP: Experiment 1; ROI: left-anterior.
(PDF)

Text S1 **Additional sentence examples for each of the critical sentence conditions.**
(PDF)
