# Supplementary figures and images for: Yes, You Can? A Speaker’s Potency to Act upon His Words Orchestrates Early Neural Responses to Message-Level Meaning
Source: PLoS One. 2013 Jul 24;8(7):e69173. doi: 10.1371/journal.pone.0069173 (PMC3722173; doi:10.1371/journal.pone.0069173)

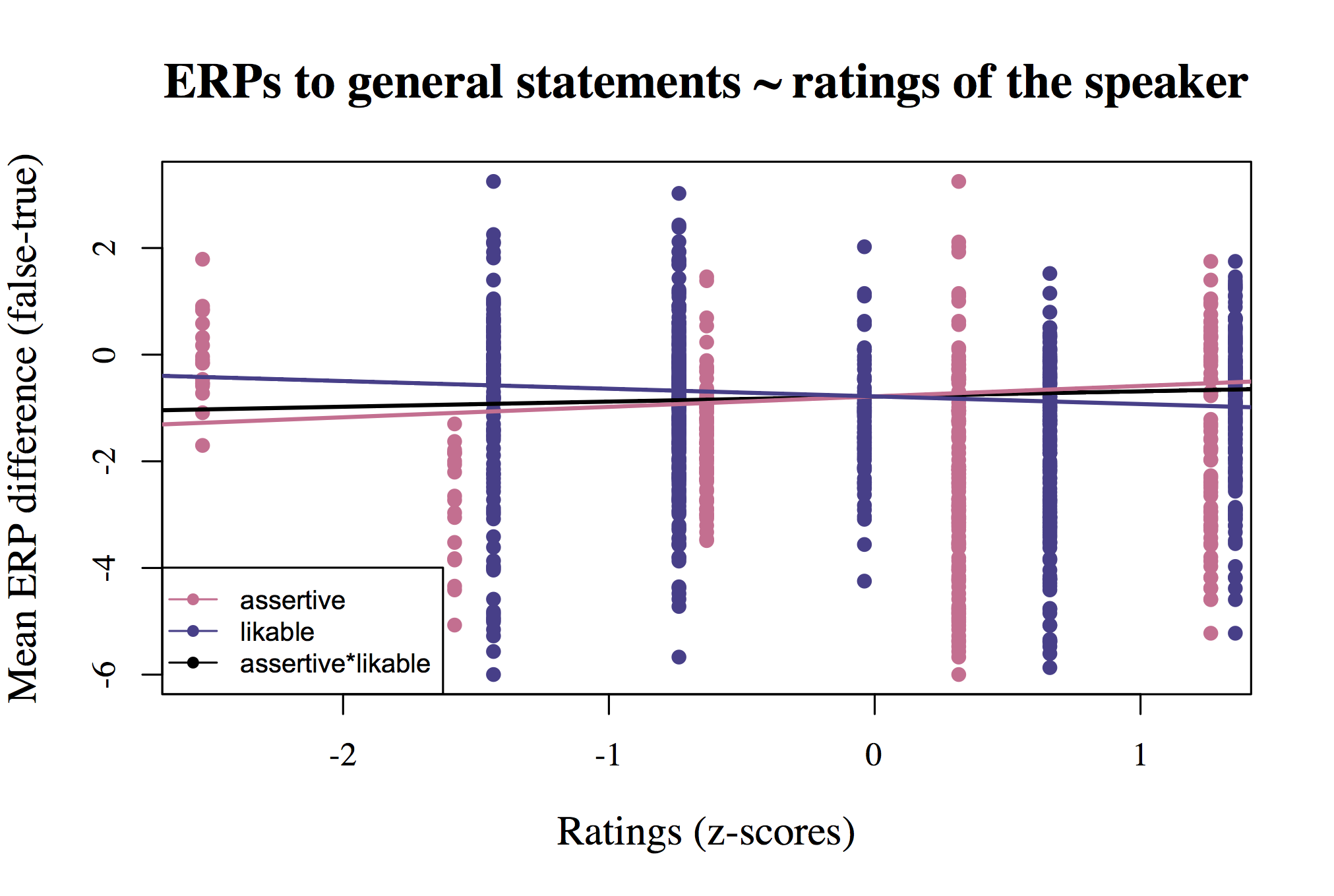

Supplement: Figure S1 — Correlations between by-participant assertiveness ratings, by-participant likability ratings and the interaction of assertiveness x likability for the political speaker and the TRUE-FALSE effect for general statements (ERP amplitudes for false - true statements in the 300–450 ms time window). In contrast to political statements (see the main text and Figures 4 and 5), N400 amplitudes for false versus true general statements did not show a correlation with the likability and assertiveness of the political speaker. (TIFF) [file pone.0069173.s001.tiff]
