# Supplementary material for: Yes, You Can? A Speaker’s Potency to Act upon His Words Orchestrates Early Neural Responses to Message-Level Meaning
Source: PLoS One. 2013 Jul 24;8(7):e69173. doi: 10.1371/journal.pone.0069173 (PMC3722173; doi:10.1371/journal.pone.0069173)
Supplement: Table S1 — Mean plausibility ratings for the critical sentence materials as determined in a questionnaire pre-test (standard deviations are given in parentheses). Ratings were obtained on a 6-point scale (1 = “highly plausible”, 6 = “completely implausible”). Statistical analysis of the ratings via a repeated measures analysis of variance (ANOVA) revealed a main effect of TRUE-FALSE (F(1,39) = 1555.73, p<0.0001) and interaction of SENTENCE-TYPE × TRUE-FALSE (F(1,39) = 142.94, p<0.0001). (Note that this analysis was performed by-participants only, since items differed across sentence types.) Simple comparisons for each sentence type were performed by-participants (F1) and by-items (F2) and are reported in the table. (PDF) [file pone.0069173.s014.pdf]

Table S1: Mean plausibility ratings for the critical sentence materials as determined in a questionnaire pre-test (standard deviations are given in parentheses). Ratings were obtained on a 6-point scale (1 = "highly plausible", 6 = "completely implausible"). Statistical analysis of the ratings via a repeated measures analysis of variance (ANOVA) revealed a main effect of TRUE-FALSE ($F(1,39) = 1555.73$, $p < 0.0001$) and interaction of SENTENCE-TYPE x TRUE-FALSE ($F(1,39) = 142.94$, $p < 0.0001$). (Note that this analysis was performed by-participants only, since items differed across sentence types.) Simple comparisons for each sentence type were performed by-participants (F_1) and by-items (F_2) and are reported in the table.

Condition		Rating	Statistical analysis
GENERAL	true	1.40 (0.32)	$F_1(1,39) = 2549.37$, $p < 0.0001$<br>$F_2(1,35) = 676.42$, $p < 0.0001$
	false	5.59 (0.39)	
POLITICAL	true	2.39 (0.59)	$F_1(1,39) = 346.02$, $p < 0.0001$<br>$F_2(1,35) = 66.44$, $p < 0.0001$
	false	4.86 (0.64)	
