# Supplementary material for: Yes, You Can? A Speaker’s Potency to Act upon His Words Orchestrates Early Neural Responses to Message-Level Meaning
Source: PLoS One. 2013 Jul 24;8(7):e69173. doi: 10.1371/journal.pone.0069173 (PMC3722173; doi:10.1371/journal.pone.0069173)
Supplement: Table S2 — Parameter values for the fixed effects in the linear mixed effects model for the first N400 time window (150–300 ms). The model was fit using a maximal random effects structure and a minimal adequate fixed effects structure (see the main text for details). For reasons of readability, only effects approaching significance (|t| >1.9) are reported. In addition, in view of the research questions pursued here, we only report effects of or interactions including TRUE-FALSE. Note that the reference levels for the fixed factors were as follows: TRUE-FALSE: false; SENTENCE-TYPE: general; SPEAKER: control; GROUP: Experiment 1; ROI: left-anterior. (PDF) [file pone.0069173.s015.pdf]

Table S2: Parameter values for the fixed effects in the linear mixed effects model for the first N400 time window (150-300 ms). The model was fit using a maximal random effects structure and a minimal adequate fixed effects structure (see the main text for details). For reasons of readability, only effects approaching significance (|t| > 1.9) are reported. In addition, in view of the research questions pursued here, we only report effects of or interactions including TRUE-FALSE. Note that the reference levels for the fixed factors were as follows: TRUE-FALSE: false; SENTENCE-TYPE: general; SPEAKER: control; GROUP: Experiment 1; ROI: left-anterior.

**Effect**	**Estimate**	**Standard error**	***t*-value**
Intercept	-1.27	0.31	-4.07
TRUE-FALSE(true)	0.88	0.40	2.21
GROUP(exp2):TRUE-FALSE(true)	-0.96	0.44	-2.16
TRUE-FALSE(true):SPEAKER(prominent): ROI(l-post)	0.80	0.35	2.40
TRUE-FALSE(true):SPEAKER(prominent): ROI(r-post)	0.77	0.34	2.28
GROUP(exp2):SPEAKER(prominent):TRUE-FALSE(true)	1.22	0.30	4.00
TRUE-FALSE(true):TYPE(political): ROI(l-post)	-0.90	0.34	-2.62
GROUP(exp2):SPEAKER(prominent):TRUE-FALSE(true): ROI(l-post)	-1.14	0.40	-2.87
GROUP(exp2):SPEAKER(prominent):TRUE-FALSE(true): ROI(r-post)	-1.27	0.39	-3.19
GROUP(exp2):TYPE(political):TRUE-FALSE(true): ROI(l-post)	1.07	0.40	2.70
GROUP(exp2):SPEAKER(prominent):TYPE(political): TRUE-FALSE(true)	-0.88	0.23	-3.83
