# Supplementary material for: Yes, You Can? A Speaker’s Potency to Act upon His Words Orchestrates Early Neural Responses to Message-Level Meaning
Source: PLoS One. 2013 Jul 24;8(7):e69173. doi: 10.1371/journal.pone.0069173 (PMC3722173; doi:10.1371/journal.pone.0069173)
Supplement: Table S3 — Parameter values for the fixed effects in the linear mixed effects model for the second N400 time window (300–450 ms). The model was fit using a maximal random effects structure and a minimal adequate fixed effects structure (see the main text for details). For reasons of readability, only effects approaching significance (|t| >1.9) are reported. In addition, in view of the research questions pursued here, we only report effects of or interactions including TRUE-FALSE. Note that the reference levels for the fixed factors were as follows: TRUE-FALSE: false; SENTENCE-TYPE: general; SPEAKER: control; GROUP: Experiment 1; ROI: left-anterior. (PDF) [file pone.0069173.s016.pdf]

Table S3: Parameter values for the fixed effects in the linear mixed effects model for the second N400 time window (300-450 ms). The model was fit using a maximal random effects structure and a minimal adequate fixed effects structure (see the main text for details). For reasons of readability, only effects approaching significance (|t| > 1.9) are reported. In addition, in view of the research questions pursued here, we only report effects of or interactions including TRUE-FALSE. Note that the reference levels for the fixed factors were as follows: TRUE-FALSE: false; SENTENCE-TYPE: general; SPEAKER: control; GROUP: Experiment 1; ROI: left-anterior.

**Effect**	**Estimate**	**Standard error**	***t*-value**
Intercept	-0.90	0.37	-2.43
TRUE-FALSE(true):ROI(l-cent)	0.68	0.26	2.60
TRUE-FALSE(true):ROI(l-post)	0.99	0.26	3.80
TRUE-FALSE(true):ROI(r-ant)	0.64	0.26	2.45
TRUE-FALSE(true):ROI(r-cent)	1.07	0.26	4.10
TRUE-FALSE(true):ROI(r-post)	0.92	0.26	3.53
TRUE-FALSE(true):TYPE(political): ROI(l-cent)	-0.78	0.34	-2.27
TRUE-FALSE(true):TYPE(political): ROI(l-post)	-1.16	0.34	-3.37
TRUE-FALSE(true):TYPE(political): ROI(r-cent)	-0.90	0.34	-2.61
TRUE-FALSE(true):TYPE(political): ROI(r-post)	-0.94	0.34	-2.73
TRUE-FALSE(true):GROUP(exp2):TYPE(political): ROI(r-post)	0.94	0.40	2.36
TRUE-FALSE(true):SPEAKER(prominent):TYPE(political): ROI(r-post)	-1.14	0.40	-2.86
GROUP(exp2):SPEAKER(prominent):TYPE(political): TRUE-FALSE(true)	-0.75	0.23	-3.22
