# Supplementary material for: Yes, You Can? A Speaker’s Potency to Act upon His Words Orchestrates Early Neural Responses to Message-Level Meaning
Source: PLoS One. 2013 Jul 24;8(7):e69173. doi: 10.1371/journal.pone.0069173 (PMC3722173; doi:10.1371/journal.pone.0069173)
Supplement: Table S4 — Parameter values for the fixed effects in the linear mixed effects model for the first late positivity time window (600–750 ms). The model was fit using a maximal random effects structure and a minimal adequate fixed effects structure (see the main text for details). For reasons of readability, only effects approaching significance (|t| >1.9) are reported. In addition, in view of the research questions pursued here, we only report effects of or interactions including TRUE-FALSE. Note that the reference levels for the fixed factors were as follows: TRUE-FALSE: false; SENTENCE-TYPE: general; SPEAKER: control; GROUP: Experiment 1; ROI: left-anterior. (PDF) [file pone.0069173.s017.pdf]

Table S4: Parameter values for the fixed effects in the linear mixed effects model for the first late positivity time window (600-750 ms). The model was fit using a maximal random effects structure and a minimal adequate fixed effects structure (see the main text for details). For reasons of readability, only effects approaching significance (|t| > 1.9) are reported. In addition, in view of the research questions pursued here, we only report effects of or interactions including TRUE-FALSE. Note that the reference levels for the fixed factors were as follows: TRUE-FALSE: false; SENTENCE-TYPE: general; SPEAKER: control; GROUP: Experiment 1; ROI: left-anterior.

**Effect**	**Estimate**	**Standard error**	***t*-value**
Intercept	0.84	0.41	2.04
TRUE-FALSE(true)	-1.04	0.45	-2.29
TRUE-FALSE(true):ROI(l-cent)	-0.97	0.21	-4.73
TRUE-FALSE(true):ROI(l-post)	-1.54	0.21	-7.49
TRUE-FALSE(true):ROI(r-cent)	-0.58	0.21	-4.73
TRUE-FALSE(true):ROI(r-post)	-1.29	0.21	-6.27
TRUE-FALSE(true):SPEAKER(prominent)	1.08	0.35	3.08
TRUE-FALSE(true):SPEAKER(prominent):ROI(l-post)	-0.51	0.21	-2.49
TRUE-FALSE(true):SPEAKER(prominent):ROI(l-post)	-0.60	0.21	-2.92
TRUE-FALSE(true):GROUP(exp2):SPEAKER(prominent)	-0.65	0.12	-5.45
TRUE-FALSE(true):TYPE(political): ROI(l-cent)	0.75	0.21	3.63
TRUE-FALSE(true):TYPE(political): ROI(l-post)	1.59	0.21	7.71
TRUE-FALSE(true):TYPE(political): ROI(r-cent)	0.64	0.21	3.13
TRUE-FALSE(true):TYPE(political): ROI(r-post)	1.54	0.21	7.47
