# Supplementary material for: Yes, You Can? A Speaker’s Potency to Act upon His Words Orchestrates Early Neural Responses to Message-Level Meaning
Source: PLoS One. 2013 Jul 24;8(7):e69173. doi: 10.1371/journal.pone.0069173 (PMC3722173; doi:10.1371/journal.pone.0069173)
Supplement: Table S5 — Parameter values for the fixed effects in the linear mixed effects model for the second late positivity time window (750–900 ms). The model was fit using a maximal random effects structure and a minimal adequate fixed effects structure (see the main text for details). Since the maximal model did not converge, the random effects structure was simplified by removing the by-participant random slope for speaker, as this was the random slope with the smallest variance (as suggested by [30]). For reasons of readability, only effects approaching significance (|t| >1.9) are reported. In addition, in view of the research questions pursued here, we only report effects of or interactions including TRUE-FALSE. Note that the reference levels for the fixed factors were as follows: TRUE-FALSE: false; SENTENCE-TYPE: general; SPEAKER: control; GROUP: Experiment 1; ROI: left-anterior. (PDF) [file pone.0069173.s018.pdf]

Table S5: Parameter values for the fixed effects in the linear mixed effects model for the second late positivity time window (750-900 ms). The model was fit using a maximal random effects structure and a minimal adequate fixed effects structure (see the main text for details). Since the maximal model did not converge, the random effects structure was simplified by removing the by-participant random slope for speaker, as this was the random slope with the smallest variance (as suggested by [30]). For reasons of readability, only effects approaching significance ($|t| > 1.9$) are reported. In addition, in view of the research questions pursued here, we only report effects of or interactions including TRUE-FALSE. Note that the reference levels for the fixed factors were as follows: TRUE-FALSE: false; SENTENCE-TYPE: general; SPEAKER: control; GROUP: Experiment 1; ROI: left-anterior.

| **Effect** | **Estimate** | **Standard error** | ***t*-value** |
|---|---|---|---|
| TRUE-FALSE(true):ROI(l-cent) | -0.81 | 0.28 | -2.93 |
| TRUE-FALSE(true):ROI(l-post) | -1.77 | 0.28 | -6.33 |
| TRUE-FALSE(true):ROI(r-cent) | -1.08 | 0.28 | -3.85 |
| TRUE-FALSE(true):ROI(r-post) | -1.68 | 0.28 | -6.02 |
| GROUP(exp2):SPEAKER(prominent):TRUE-FALSE(true) | -1.36 | 0.40 | -3.42 |
| TRUE-FALSE(true):TYPE(political): ROI(l-post) | 1.25 | 0.40 | 3.15 |
| TRUE-FALSE(true):GROUP(exp2):SPEAKER(prominent): ROI(l-post) | 1.27 | 0.56 | 2.26 |
| GROUP(exp2):SPEAKER(prominent):TYPE(political): TRUE-FALSE(true) | 1.50 | 0.57 | 2.66 |
| GROUP(exp2):SPEAKER(prominent):TYPE(political): TRUE-FALSE(true):ROI(r-post) | -1.51 | 0.80 | -1.90 |
