# Supplementary material for: Yes, You Can? A Speaker’s Potency to Act upon His Words Orchestrates Early Neural Responses to Message-Level Meaning
Source: PLoS One. 2013 Jul 24;8(7):e69173. doi: 10.1371/journal.pone.0069173 (PMC3722173; doi:10.1371/journal.pone.0069173)
Supplement: Text S1 — Additional sentence examples for each of the critical sentence conditions. (PDF) [file pone.0069173.s019.pdf]

Additional sentence examples for each of the critical sentence conditions

Examples are accompanied by a literal (word-by-word) translation. A full list of materials can be obtained from the corresponding author upon request.

POLITICAL - TRUE

Die Bundesregierung fördert in Ostdeutschland zukunftsorientierte Forschungsprojekte.
the Federal Government supports in East Germany future-oriented research projects

Das statistische Bundesamt meldet für das erste Quartal 2007 ein 1.5 prozentiges Wirtschaftswachstum.
the Federal Statistics Office announces for the first quarter 2007 a 1.5 percent economic growth

Das Bundesamt für Verfassungsschutz schafft ein Aussteigerprogramm für Rechtsextremisten.
the Federal Office for the Protection of the Constitution is creating an exit programme for right-wing extremists

Das Ministerium für Ernährung erarbeitet neue Standards für die Schulspeisung.
the Ministry of Food is developing new standards for school meals

Ein großes Thema des Außenministeriums bleibt die Bekämpfung des Terrorismus.
a big topic of the Foreign Ministry remains the fight against terrorism

POLITICAL - FALSE

Das Bundesforschungsministerium verbietet alle deutschen Forschungsprojekte.
the Federal Ministry of Science prohibits all German research projects

Die Bundesregierung zielt in der Zukunft auf eine Volkswirtschaft ohne Wirtschaftswachstum.
the Federal Government aims in the future for an economy without economic growth

Das Bundespresseamt berät die junge Führungselite der Rechtsextremisten
the Federal Press Office advises the young leadership of the right-wing extremists

Das Bildungsministerium bewilligt genmanipulierte Lebensmittel für die Schulspeisung
the Ministry of Education appoves genetically manipulated food for school meals

Die Bundesregierung fördert in Sachsen den radikalen Terrorismus
the Federal Government supports in Saxony radical terrorism

GENERAL - TRUE

Minnie ist die Freundin von Micky Maus.
Minnie is the girlfriend of Micky Mouse

Peking ist die Hauptstadt von China.
Beijing is the capital of China

Theologie ist die Lehre von den Religionen.
theology is the study of the religions

Die Cheops-Pyramide findet man in Ägypten.
the Pyramid of Cheops finds one in Egypt

Der Louvre in Paris dient der Präsentation von Gemälden.
the Louvre in Paris serves the presentation of paintings
